# Voxel-Based Texture Analysis of the Brain

**DOI:** 10.1371/journal.pone.0117759

**Published:** 2015-03-10

**Authors:** Rouzbeh Maani, Yee Hong Yang, Sanjay Kalra

**Affiliations:** 1 Department of Computing Science, University of Alberta, Edmonton, Canada; 2 Departments of Medicine, Computing Science, and Biomedical Engineering, University of Alberta, Edmonton, Canada; Bellvitge Biomedical Research Institute-IDIBELL, SPAIN

## Abstract

This paper presents a novel voxel-based method for texture analysis of brain images. Texture analysis is a powerful quantitative approach for analyzing voxel intensities and their interrelationships, but has been thus far limited to analyzing regions of interest. The proposed method provides a 3D statistical map comparing texture features on a voxel-by-voxel basis. The validity of the method was examined on artificially generated effects as well as on real MRI data in Alzheimer's Disease (AD). The artificially generated effects included hyperintense and hypointense signals added to T1-weighted brain MRIs from 30 healthy subjects. The AD dataset included 30 patients with AD and 30 age/sex matched healthy control subjects. The proposed method detected artificial effects with high accuracy and revealed statistically significant differences between the AD and control groups. This paper extends the usage of texture analysis beyond the current region of interest analysis to voxel-by-voxel 3D statistical mapping and provides a hypothesis-free analysis tool to study cerebral pathology in neurological diseases.

## Introduction

Texture analysis is a powerful image analysis method that quantitates voxel intensities (or pixel intensities in 2D) and their patterns and interrelationships. Texture analysis can identify intensity patterns including those that cannot easily be detected by the unaided human eye [1]. Applied to MR images, the methods have been successfully used to study several neurological diseases including brain tumor [2–3], epilepsy [4–6], Alzheimer’s disease [7–8], and multiple sclerosis [9–11]. Robustness to MRI acquisition parameters [12] and noise [13–15] makes texture analysis a reliable and attractive tool for investigation of neuropsychiatric conditions. However, current texture analysis methods are limited to region of interest (ROI) based analysis and require a priori hypotheses directing the analysis to specific brain regions.

An alternative approach to ROI analysis is the hypothesis free method in which regions with significant statistical difference are automatically detected between groups. One of the most popular examples of this type of analysis is voxel based morphometry (VBM) [16], a technique that performs a voxel-based statistical analysis on gray (GM) or white matter (WM) density.

Inspired by VBM, a novel method to perform texture analysis in a voxel-based manner is presented in this paper. The output of the proposed method is a statistical map, similar to that of VBM, indicating regions with statistically significant differences. However, a texture feature, instead of the amount of GM or WM, is compared at each voxel.

The proposed method is validated on a dataset with artificially generated lesions and on one of Alzheimer’s disease (AD). This proposed extension to texture analysis provides a powerful tool for investigation of brain MRIs in different neurological diseases.

## Materials

To examine the validity of the proposed method, two different approaches were used. First, an MRI dataset with artificial effects was generated. Second, a dataset of healthy subjects and patients with AD was used. AD was chosen because the spatial distribution of pathological changes in the brain is well known in this disease. Both datasets were derived from the OASIS database (http://www.oasis-brains.org) [17] which includes a collection of 416 right-handed healthy controls and patients with early-stage AD and accompanying 3D T1-weigthted magnetization prepared rapid gradient echo (MPRAGE) images acquired at 1.5 tesla (repetition time [TR] = 9.7 ms, echo time [TE] = 4.0 ms, inversion time [TI] = 20 ms, flip angle = 10°, orientation = sagittal). The images include 128 slices (slice thickness = 1.25 mm, in-plane resolution 1.0 × 1.0 mm^2^) covering the whole brain. The MRI protocol was the same for all subjects (see [17] for details).

The criterion to choose subjects from the OASIS database was the Clinical Dementia Rating (CDR) score. For our experiments we chose all subjects diagnosed with CDR of 1 (mild AD) or 2 (moderate AD). This resulted in a dataset of 30 subjects in total, with a gender distribution of 20 females and 10 males. The average age was 78 ± 7 years. A group of healthy control subjects from the OASIS database were selected that were matched for age and gender of the subjects with AD.

The database of artificial effects was created as the ground truth to validate the proposed method. MRIs from the selected healthy control subjects were used for this purpose. Two types of artifacts were added: hyper-intense and hypo-intense. For each type of artifact, 60 locations in the brain were chosen (30 in each hemisphere, S1 Fig.), with varying size and Gaussian signal properties (S1 Table). The 60 locations included regions that included pure GM, pure WM and mixed GM/WM (border of GM/WM).

## Methods

The processing pipeline of the proposed method includes three main parts: pre-processing, texture feature computation, and voxel-based statistical analysis. The first and the last parts have been provided by several medical image analysis tools. The second part is the core of the proposed method and has been developed as a toolbox which can be easily integrated with other brain analysis tools easily. The next subsections explain each part in details.

### Image Pre-Processing

The pre-processing part of the pipeline includes two main steps. The first step is to normalize images into a template atlas such that a voxel-base analysis can be performed. The second step of preprocessing is correcting non-uniformity variations and intensity standardization which makes the intensity of the images between subjects comparable. The preprocessing steps were performed using the VBM8 toolbox (http://dbm.neuro.uni-jena.de/vbm/) with default parameters. The VBM8 toolbox is an extension of the unified segmentation model [18] using the high-dimensional DARTEL procedure [19] to normalize images to the MNI152 atlas.

### 3D Texture Analysis

Texture refers to the intensity variations or visual patterns in images. Indeed, how we perceive an image is not limited to intensity per se. The human eye is able to distinguish different objects and scenes by means of visual patterns or textures, such as smoothness, coarseness, and regularity.

There are different techniques to defining and quantifying textures [1]. A popular approach is to extract (calculate) the statistical relationship between neighboring pixels (or voxels in 3D). The extracted statistical information is used to form what is known as a texture feature.

In this paper, a well-known statistical texture analysis method, the 2D gray level co-occurrence matrix (GLCM) [20], is extended to define texture features on a voxel-by-voxel basis in 3D images.

In mathematical formation a grayscale image, *I*, is a matrix of numbers which represent intensities in the image. Assume that these numbers (intensities or gray levels) range from 1 to *N*_*g*_ and the image has a height of *N*_*x*_ and a width of *N*_*y*_. *N*_*g*_ is known as the quantization level. S2(a) Fig. shows an example of an image with *N*_*g*_ = 3, *N*_*x*_ = 4, and *N*_*y*_ = 4. By considering the above mentioned assumptions, the image *I* is represented as a function mapping the spatial domain to the gray values:

$$I:L_{y}\times L_{x}\to G$$ (1)

where L_y_ = {1,…,N_y_} denotes the spatial domain along the *y* axis, L_x_ = {1,…,N_x_} the spatial domain along the *x* axis, and G = {1,. . .,N_g_} the gray values. Here, the Haralick notation [20] was followed which assigns the y axis to the first dimension. Note that, the quantization level can be changed to a specific value. Assume that the target quantization level is *N*_*g*_ and the original quantization level of the image is Q_g_(G = {1,. . .,Q_g_}). The image is transformed into the target quantization level by

$$I(x,y)=round\left(\frac{\left(I(x,y)-1\right)}{\left(Q_{g}-1\right)}(N_{g}-1)\right)+1$$ (2)

where I(x,y) is the intensity value of the pixel in location (x,y), and *round* is the round function which returns the closest integer value of the given non-integer number.

To define GLCM, first an offset should be defined. On a 2D plane, an offset with distance *d* and direction angle *Θ* is represented by *O = [a*, *b] = [d sin(Θ)*, *d cos(Θ)]* connecting pixel *I(k*, *l)* to pixel *I(m*,*n)* such that *m = k+a* and n = l+b. For instance, an offset with distance of 1 and angle of 90^o^ increases *m* by 1 and *n* by 0 or offsets them from the original position with [10] (S2(b) Fig.). GLCM_O_ is defined for the specific offset *O = [a*,*b]* as follows:

$$\begin{array}{l} GLCM_{O}(i,j)=\#\{\left(\left(k,l),(m,n\right)\right)\in (L_{y}\times L_{x})\times (L_{y}\times L_{x})|\\ \;m=k+a,n=l+b,I(k,l)=i,I(m,n)=j\}. \end{array}$$ (3)

In other words, the GLCM for a specific offset is an N_g_ *×* N_g_ matrix where the entry *(i*, *j)* shows the number of times that *I(k*,*l) = i* and *I(m*,*n) = j*. S2 Fig. explains the construction of GLCM for a sample image. GLCM is easily extendable to 3D by considering offsets in a 3D space [21]. Formally, a 3D image with *G* gray levels is defined as:

$$I:L_{y}\times L_{x}\times L_{z}\to G,$$ (4)

$$\begin{array}{l} GLCM_{O}(i,j)=\#\{\left(\left(k,l,u),(m,n,v\right)\right)\in (L_{y}\times L_{x}\times L_{z})\times (L_{y}\times L_{x}\times L_{z})|\\ \;m=k+a,n=l+b,v=u+c,I(k,l,u)=i,I(m,n,v)=j\}, \end{array}$$ (5)

where L_z_ = {1,…,N_z_} denotes the spatial domain along the *z* axis and the offset O connects voxel I(k,l,u) to I(m,n,v). In the traditional GLCM, the texture features are computed for a region of interest while the goal of the proposed method is to find texture features at each voxel. To do this, a spherical volume of radius *R* is considered around each voxel. Formally, the voxel-based GLCM in 3D (“VGLCM-3D”) is defined for a specific neighborhood of radius R, and offset O = [a, b, c] for the voxel V located at (Vy, Vx, Vz):

$$\begin{array}{l} VGLCM-3D_{R,O}(i,j)=\#\{\left(\left(k,l,u),(m,n,v\right)\right)\in S^{R}(V_{y},V_{x},V_{z})\times S^{R}(V_{y},V_{x},V_{z})|\\ \;m=k+a,n=l+b,v=u+c,I(k,l,u)=i,I(m,n,v)=j\}, \end{array}$$ (6)

where *S* ^*R*^(*V*_*y*_, *V*_*x*_, *V*_*z*_) denotes the neighborhood region with a radius of *R* around each voxel:

$$\begin{array}{l} S^{R}(V_{y},V_{x},V_{z})=\{(y,x,z)|y=\{1,...,N_{y}\},x=\{1,...,N_{x}\},z=\{1,...,N_{z}\},\\ \;\sqrt{{\left(y-V_{y}\right)}^{2}+{\left(x-V_{x}\right)}^{2}+{\left(z-V_{z}\right)}^{2}}\leq R\}. \end{array}$$ (7)

One may note the difference of the proposed VGLCM-3D with traditional GLCM by comparing Eq. (5) and Eq. (6). Indeed in the proposed method the co-occurrence matrix is computed in a sphere of radius R around each voxel instead of the whole region of interest.

To compute texture features at each voxel, first the co-occurrence matrices for all offsets with distance of *d* are computed at each voxel by Eq. (6). After computing GLCMs for all offsets, they are summed over all offsets and normalized (i.e., divided by the sum):

$$VGLCM-3D_{R}^{sum}(i.j)=\sum_{\forall O}^{}VGLCM-3D_{R,O}^{}(i.j)$$ (8)

$$VGLCM-3D_{R}^{norm}(i.j)=\frac{VGLCM-3D_{R}^{sum}(i.j)}{\sum_{i=1}^{N_{g}}\sum_{j=1}^{N_{g}}VGLCM-3D_{R}^{sum}(i.j)}$$ (9)

Eight texture features were computed for analysis. The list of the features is given in S2 Table. The value of the $VGLCM-3D_{R}^{norm}$ computed by (Equation 9) is used as the probability function *p* to define the features in S2 Table. Additional texture features could also be computed [1]. Nonetheless, these features are enough to show the capability of texture analysis.

It is notable that the computational expense for 3D analysis increases rapidly as distance *d* increases. For distance of *d* there are (2d+1)^2^–1 offsets in 2D and (2d+1)^3^–1 offsets in 3D. For example, for a distance of 1 there are 8 possible offsets in 2D (i.e., {[-1,-1],[-1,0],…,[1,1]}) while there are 26 offsets in 3D (i.e., {[-1,-1,-1],[-1,-1,0],…,[1,1,1]}). For a distance of d≤2 there are 24 offsets in 2D while there are 124 offsets in 3D.

To alleviate the computational expense, in addition to VGLCM-3D, a less computationally-expensive approach called voxel-based GLCM on three orthogonal planes in 3D space (“VGLCM-TOP-3D”) is proposed. Herein GLCM is computed individually in the axial, coronal, and sagittal planes at each voxel and the final feature value is the average of these 3 texture values in the three planes. Using this approach there will be 3×((2d+1)^2^–1) offsets for computation. For instance, this approach will use 24 offsets for d = 1 and 72 offsets for d≤2 (compared to 26 and 124 offsets for VGLCM-3D), reducing the number of offsets considerably for large distances. Assuming that the *z* axis refers to the Up-Down direction, *x* axis the Left-Right, and *y* axis the Anterior-Posterior in an MRI image, the axial, sagittal, and coronal planes are defined as:

$$I_{axi}(k,l,u)=\left\{I(y,x,z)|y=\{1,...,N_{y}\},x=\{1,...,N_{x}\},z=u\right\}$$ (10)

$$I_{sag}(k,l,u)=\left\{I(y,x,z)|y=\{1,...,N_{y}\},z=\{1,...,N_{z}\},x=l\right\}$$ (11)

$$I_{cor}(k,l,u)=\left\{I(y,x,z)|x=\{1,...,N_{x}\},z=\{1,...,N_{z}\},y=k\right\}$$ (12)

where u = {1,…,N_z_}, l = {1,…,N_x_}, and k = {1,…,N_y_} denote the location of the slice in the axial, sagittal, and coronal views, respectively. VGLCM-TOP-3D is defined for a specific plane *P* with a neighborhood radius of *R* and offset *O = [a*,*b*,*c]* for voxel V located at (Vy, Vx, Vz):

$$\begin{array}{l} VGLCM-TOP-3D_{P,R,O}(i,j)=\#\{\left(\left(k,l,u),(m,n,v\right)\right)\in S_{P}^{R}(V_{y},V_{x},V_{z})\times S_{P}^{R}(V_{y},V_{x},V_{z})|\\ \;m=k+a,n=l+b,v=u+c,I_{P}(k,l,u)=i,I_{P}(m,n,v)=j\} \end{array}$$ (13)

where *I*_*P*_ is defined by Equations. 10, 11, 12, and S_P_^R^is defined for the axial, sagittal, and coronal planes, respectively:

$$S_{axi}^{R}(V_{y},V_{x},V_{z})=\{(y,x,z)|y=\{1,...,N_{y}\},x=\{1,...,N_{x}\},z=V_{z},\sqrt{{\left(y-V_{y}\right)}^{2}+{\left(x-V_{x}\right)}^{2}}\leq R\}$$ (14)

$$S_{sag}^{R}(V_{y},V_{x},V_{z})=\{(y,x,z)|y=\{1,...,N_{y}\},z=\{1,...,N_{z}\},x=V_{x},\sqrt{{\left(y-V_{y}\right)}^{2}+{\left(z-V_{z}\right)}^{2}}\leq R\}$$ (15)

$$S_{cor}^{R}(V_{y},V_{x},V_{z})=\{(y,x,z)|x=\{1,...,N_{x}\},z=\{1,...,N_{z}\},y=V_{y},\sqrt{{\left(x-V_{x}\right)}^{2}+{\left(z-V_{z}\right)}^{2}}\leq R\}$$ (16)

**Fig 1:**
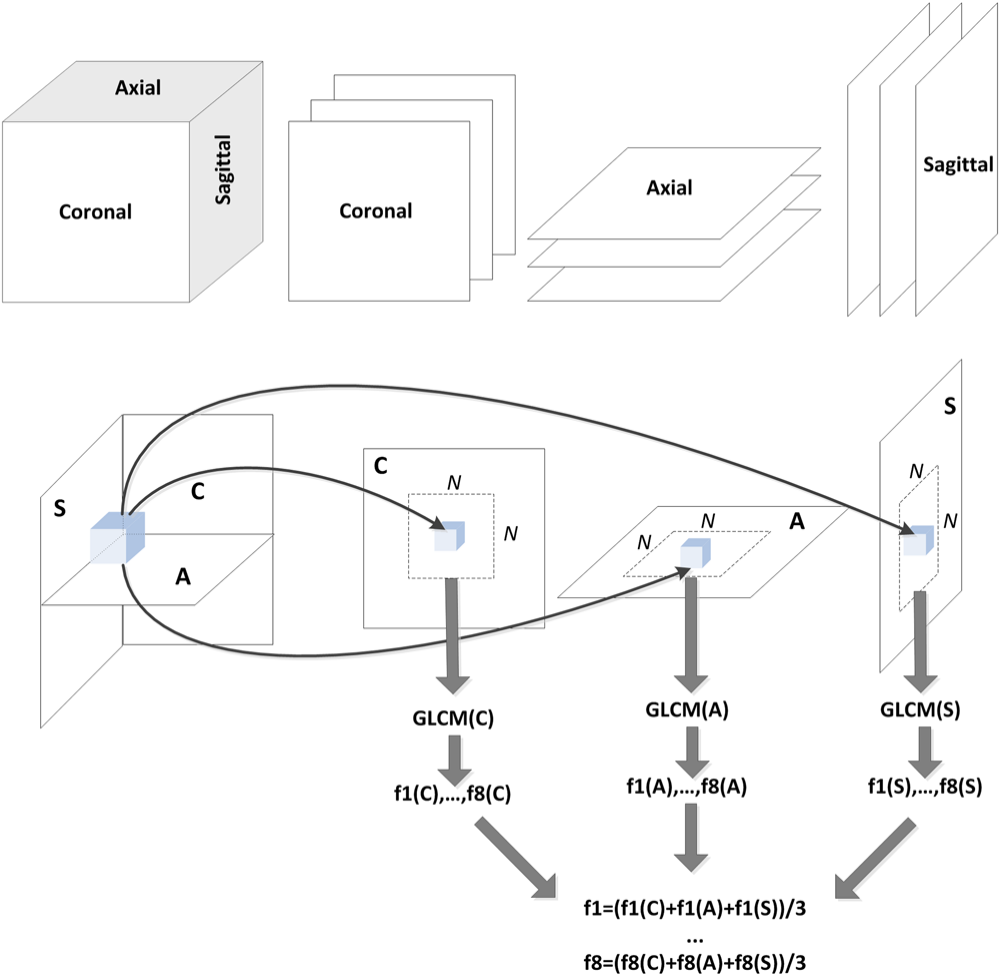
Derivation of VGLCM-TOP-3D texture features. Analysis for a voxel is performed in 3 orthogonal planes: Coronal (C), Axial (A), and Sagittal (S). Texture features (f1,… f8) are computed within a N×N region within each plane (GLCM(C), GLCM(A), GLCM(S)). The final texture is the average of the 3 local textures in each plane.

**Fig 2:**
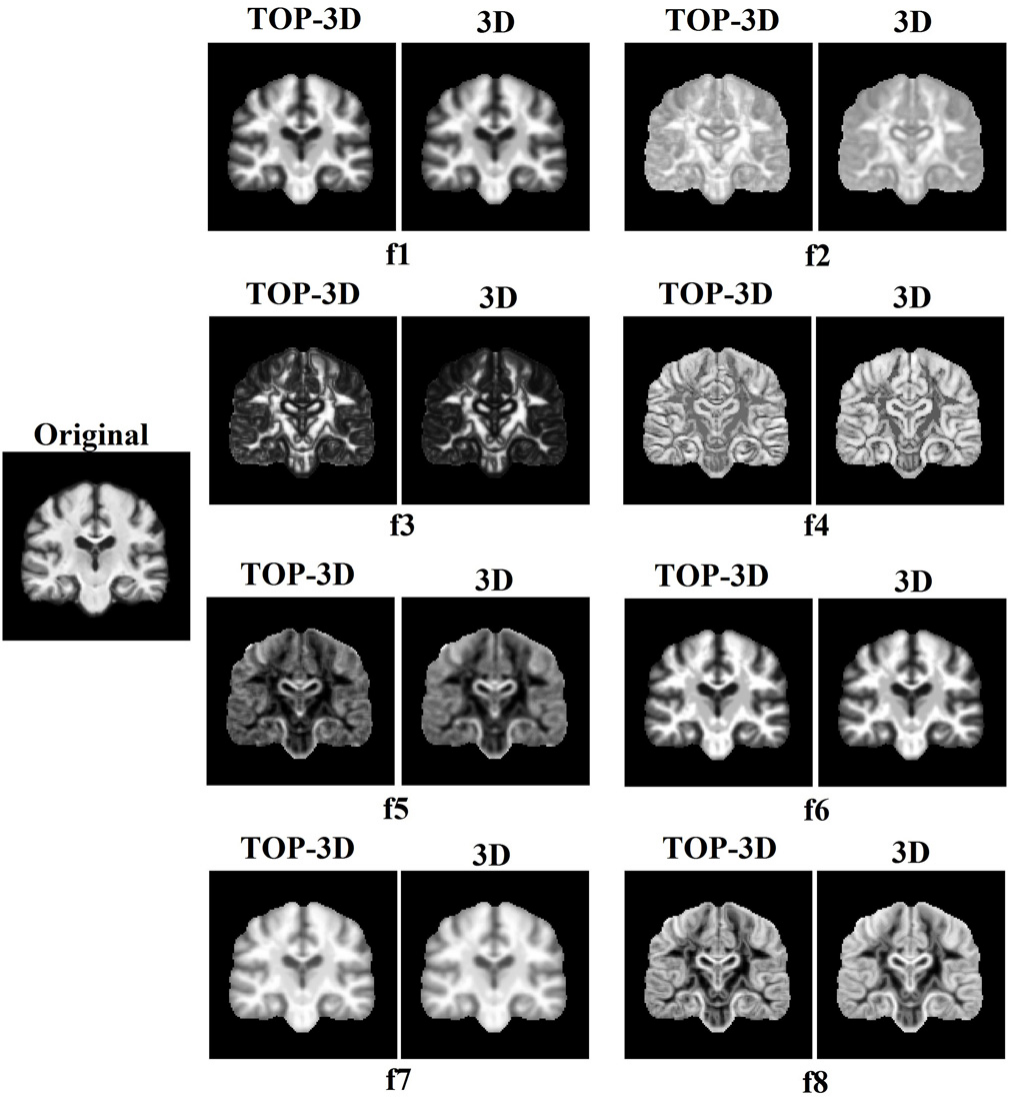
Texture Maps. Example of texture features computed by VGLCM-TOP-3D and VGLCM-3D on a sample coronal image: autocorrelation (f1), homogeneity (f2), energy (f3), correlation (f4), dissimilarity (f5), sum of squares: variance (f6), sum average (f7), and sum entropy (f8).

Similar to VGLCM-3D, the VGLCM-TOP-3D obtained for each plane is summed over all offsets and normalized. Now, at each voxel three GLCMs have been computed corresponding to the axial, sagittal, and coronal planes (i.e., $VGLCM-TOP-3D_{axi,R}^{norm}$, $VGLCM-TOP-3D_{sag,R}^{norm}$ and$VGLCM-TOP-3D_{cor,R}^{norm}$). In this step the actual texture feature is separately computed for each plane. The final texture feature is obtained as the average of that feature computed in the axial, sagittal, and coronal GLCMs. This process is illustrated in Fig. 1. The results of the texture feature computation can be visualized as texture maps (Fig. 2) that are subsequently subjected to a voxel-based statistical analysis.

### Statistical Analysis

Voxel-based statistical analysis was performed using SPM8, with the use of an F-test to produce statistical parametric maps. The F-test was used instead of the T-test as texture features of the control group could have higher or lower values compared to that of the healthy subjects. False discovery rate (FDR) correction (p<0.05) was applied to correct for multiple comparisons. To exclude the effect of age and gender in database of AD, these two factors were considered as covariates.

### Evaluation

**Fig 3:**
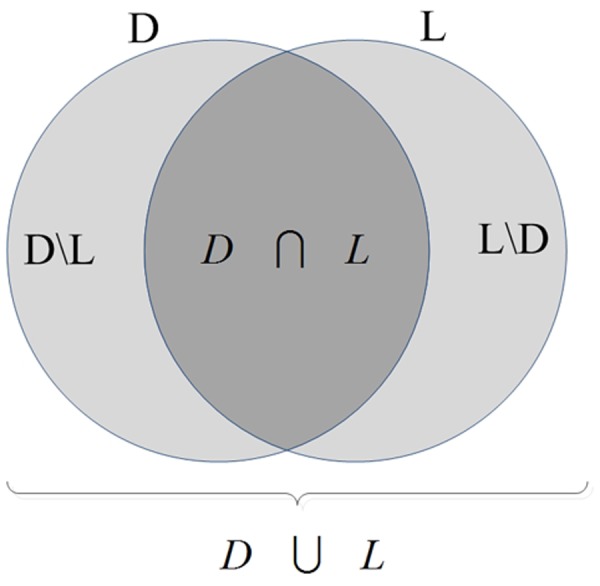
Schematic Venn diagram illustrating different possible regions considered for a detected region and an artificial lesion.

To evaluate the validity of the proposed voxel-based texture analysis a database of artificial effects was used. In addition to detection rate which shows what percentages of the artificial lesions are correctly identified, three extra measurements were determined: Jaccard coefficient, false negative error, and false positive error. The schematic Venn diagram in Fig. 3 is used to illustrate the derivation of these measures. Assume that texture features detect region “D” as the lesion while the exact lesion region is “L”. The voxels that are in “D” but not in “L” are denoted by “D\L” and the voxels that are in “L” but not in “D” are denoted by “L\D”.

The first quality measurement used in our experiments is the “union overlap,” UO [22], or the Jaccard coefficient, the intersection over the union:

$$UO=\frac{\sum_{r}\left|L_{r}\cap D_{r}\right|}{\sum_{r}\left|L_{r}\cup D_{r}\right|}$$

where *r* denotes the artificial lesions (r = {1,…,60}). This measurement indicates how well the detected regions represent the location and extension of the lesions. A lesion detection occurs when *L*_*r*_ ∩ *D*_*r*_ is not empty.

The next measurement is the false negative (FN) error [22]:

$$FN=\frac{\sum_{r}\left|L_{r}\backslash D_{r}\right|}{\sum_{r}\left|L_{r}\right|}$$

This measurement represents how much of the lesions are incorrectly found as non-lesion. Finally, the false positive (FP) error is defined [22]:

$$FP=\frac{\sum_{r}\left|D_{r}\backslash L_{r}\right|}{\sum_{r}\left|D_{r}\right|}$$

This measurement represents how much of the detected regions are incorrectly found as lesion. These measurements were computed for all 8 artificial effect types (each type included 60 artificial lesions). To better compare the effect of quantization level and the method of texture computation (VGLCM-TOP-3D vs VGLCM-3D) the measurements underwent statistical analysis using two-tailed T-test (p<0.05). For instance, UO is first computed for all lesions and all lesion types (total of 480 = 8x60 values) by quantization level of 8 and then by quantization level of 16. Next, the UO values computed by quantization level of 8 are compared with the UO values computed by quantization level of 16 using T-test to find out if one quantization level provides a statistically higher UO.

## Results

### Database of Artificial Effects

The 8 texture features shown in S2 Table have been computed using the voxel-based VGLCM-TOP-3D and VGLCM-3D method for two quantization levels of 8 and 16. Among these features f6 (Sum of squares: variance) has the best performance. Table 1 shows the performance (i.e. detection rate, union overlap, false negative and false positive errors) of this feature computed by VGLCM-TOP-3D and VGLCM-3D. This feature (f6) achieves a 100% correct detection rate in all types of artificial lesions. The last row for each method shows the average over all artificial lesion types. The highest UO and the lowest FN and FP errors are 0.67, 0.07, and 0.23, respectively. The performances of the other features are shown in S3–S9 Tables. The statistical significance of quantization level is denoted by ‡ and the statistical significance of the method (VGLCM-TOP-3D vs VGLCM-3D) is denoted by * (p<0.05). For instance, UO in VGLCM-TOP-3D with Q = 8 is statistically better (i.e., higher) than VGLCM-3D with Q = 8, while the FN Error is statistically better (i.e., lower) in VGLCM-3D with Q = 16 compared to VGLCM-3D with Q = 8.

**Table 1:** The performance of the best texture feature, f6 (Sum of squares: variance) computed for the 8 artificial effect types.

		Q = 8				Q = 16			
	Type	Detect	UO	FN Error	FP Error	Detect	UO	FN Error	FP Error
VGLCM-TOP-3D	I	100%	0.72±0.25	0.18±0.27	0.10±0.15	100%	0.71±0.25	0.16±0.26	0.13±0.19
	II	100%	0.66±0.26	0.30±0.28	0.05±0.11	100%	0.72±0.26	0.18±0.27	0.11±0.17
	III	100%	0.60±0.19	0.01±0.04	0.39±0.21	100%	0.56±0.20	0.02±0.05	0.43±0.22
	IV	100%	0.61±0.19	0.00±0.02	0.39±0.20	100%	0.65±0.22	0.00±0.01	0.35±0.22
	V	100%	0.76±0.19	0.13±0.21	0.12±0.13	100%	0.71±0.19	0.12±0.21	0.18±0.17
	VI	100%	0.80±0.18	0.13±0.19	0.08±0.10	100%	0.78±0.19	0.09±0.18	0.13±0.16
	VII	100%	0.61±0.16	0.01±0.02	0.39±0.17	100%	0.57±0.17	0.01±0.03	0.42±0.18
	VIII	100%	0.68±0.17	0.00±0.01	0.32±0.17	100%	0.70±0.20	0.00±0.01	0.30±0.20
	ALL	100%	0.67±0.21*	0.10±0.20	0.23±0.22*	100%	0.67±0.22*	0.07±0.18	0.25±0.23*
VGLCM-3D	I	100%	0.64±0.23	0.23±0.27	0.14±0.18	100%	0.61±0.22	0.20±0.27	0.21±0.20
	II	100%	0.57±0.24	0.37±0.28	0.08±0.13	100%	0.63±0.25	0.21±0.28	0.18±0.20
	III	100%	0.52±0.15	0.03±0.07	0.45±0.20	100%	0.48±0.15	0.04±0.09	0.49±0.20
	IV	100%	0.51±0.16	0.01±0.03	0.49±0.17	100%	0.56±0.19	0.01±0.02	0.44±0.20
	V	100%	0.68±0.16	0.14±0.20	0.20±0.15	100%	0.62±0.17	0.14±0.21	0.26±0.18
	VI	100%	0.72±0.16	0.17±0.19	0.12±0.13	100%	0.69±0.17	0.12±0.19	0.21±0.17
	VII	100%	0.54±0.15	0.01±0.04	0.45±0.17	100%	0.53±0.15	0.02±0.04	0.46±0.17
	VIII	100%	0.58±0.15	0.01±0.02	0.42±0.16	100%	0.61±0.17	0.01±0.02	0.38±0.17
	ALL	100%	0.59±0.19	0.12±0.21	0.29±0.23‡	100%	0.59±0.19	0.09±0.19‡	0.33±0.22

For each artificial effect type 60 artificial lesions were generated. The statistical significance of quantization level is shown by

‡ and the statistical significance of method (VGLCM-TOP-3D vs VGLCM-3D) is shown by

* (p<0.05).

Statistical comparison of the rates reveals that the UO and FP errors have a better performance using the VGLCM-TOP-3D for computation. It can also be observed that the VGLCM-3D does not outperform VGLCM-TOP-3D in any performance measurement.

With VGLCM-3D, a quantization level of 8 provides a lower FP error while a quantization level of 16 gives a lower FN error. The detection rate does not change significantly by changing the quantization level in either of the methods.

To further compare the VGLCM-TOP-3D and the VGLCM-3D methods the average performance of all 8 texture features are shown in Table 2. Similar to f6 the texture features computed by VGLCM-TOP-3D provide significantly higher UO and lower FP error compared to the features obtained by VGLCM-3D. On the other hand the FN error is significantly lower in the features computed by VGLCM-3D at Q = 16. Similar to f6, the overall FN error of features is lower at Q = 16 while their FP error is less at Q = 8 for VGLCM-TOP-3D. In addition, FN error is lower at Q = 16 compared to Q = 8 for VGLCM-TOP-3D. Similar to f6, the detection rate does not significantly change by changing the quantization level for either method.

**Table 2:** The average performance of the all features computed for the 8 artificial effect types.

		Q = 8				Q = 16			
	Type	Detect	UO	FN Error	FP Error	Detect	UO	FN Error	FP Error
VGLCM-TOP-3D	I	99%	0.48±0.27	0.35±0.31	0.31±0.25	99%	0.48±0.23	0.28±0.30	0.34±0.25
	II	89%	0.42±0.29	0.50±0.34	0.18±0.23	94%	0.46±0.28	0.36±0.37	0.27±0.25
	III	100%	0.41±0.18	0.13±0.22	0.56±0.20	100%	0.41±0.18	0.12±0.21	0.56±0.20
	IV	100%	0.42±0.19	0.14±0.24	0.54±0.20	99%	0.45±0.22	0.19±0.28	0.47±0.22
	V	100%	0.47±0.25	0.37±0.29	0.33±0.22	100%	0.48±0.22	0.29±0.29	0.36±0.21
	VI	99%	0.50±0.27	0.34±0.32	0.29±0.22	100%	0.49±0.25	0.31±0.31	0.34±0.22
	VII	100%	0.42±0.17	0.14±0.22	0.54±0.16	100%	0.42±0.16	0.13±0.22	0.54±0.17
	VIII	98%	0.45±0.23	0.27±0.31	0.40±0.25	99%	0.46±0.23	0.24±0.30‡	0.41±0.24
	ALL	99%	0.48±0.27*	0.35±0.31	0.31±0.25*	99%	0.48±0.23*	0.28±0.30	0.34±0.25*
VGLCM-3D	I	98%	0.43±0.23	0.36±0.30	0.36±0.25	98%	0.42±0.21	0.29±0.30	0.42±0.26
	II	89%	0.35±0.25	0.53±0.34	0.25±0.27	94%	0.39±0.24	0.37±0.36	0.36±0.28
	III	100%	0.34±0.15	0.11±0.18	0.64±0.17	100%	0.33±0.15	0.09±0.17	0.64±0.17
	IV	100%	0.36±0.17	0.13±0.23	0.60±0.19	99%	0.39±0.20	0.16±0.25	0.56±0.22
	V	100%	0.42±0.21	0.36±0.28	0.43±0.21	100%	0.42±0.17	0.28±0.27	0.47±0.19
	VI	99%	0.45±0.23	0.33±0.31	0.37±0.24	99%	0.44±0.21	0.28±0.29	0.43±0.22
	VII	100%	0.37±0.14	0.11±0.18	0.62±0.14	100%	0.36±0.14	0.09±0.16	0.62±0.14
	VIII	100%	0.39±0.17	0.12±0.21	0.58±0.17	100%	0.41±0.19	0.18±0.23	0.55±0.19
	ALL	98%	0.39±0.20	0.26±0.30	0.48±0.25‡	99%	0.39±0.20	0.22±0.28* ‡	0.50±0.24

For each artificial effect type 60 artificial lesions were generated. The statistical significance of quantization level is shown by

‡ and the statistical significance of method (VGLCM-TOP-3D vs VGLCM-3D) is shown by

* (p<0.05).

**Fig 4:**
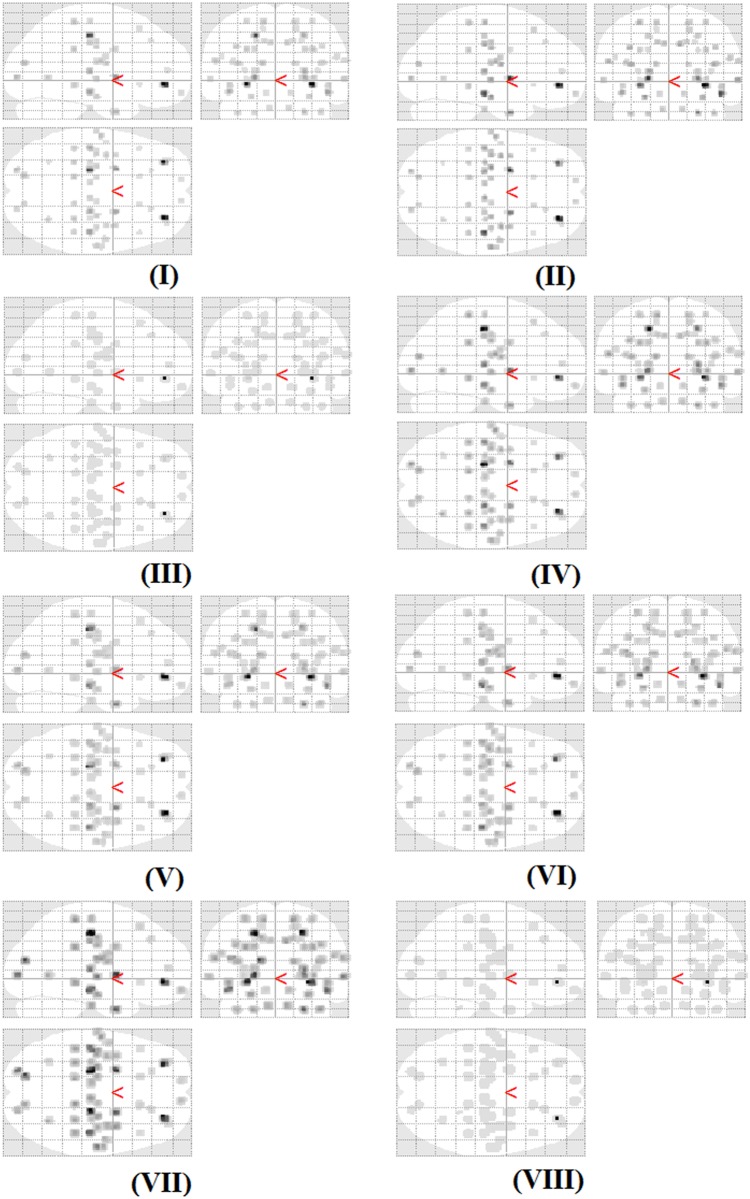
The statistical maps of sum of squares: variance (f6) in the analysis of artificial lesions. The regions with statistically significant difference (corrected by FRD at p<0.05) are shown (i.e., detection regions). The ground truth locations of lesions are shown in S1 Fig. The types of artificial lesion are shown by roman number (i.e., I, II,…,VIII) and defined in S1 Table. The maps were computed by VGLCM-TOP-3D at quantization level of 8.

The statistical maps of f6 for the 8 different artificial effects computed by VGLCM-TOP-3D at quantization level of 8 (Fig. 4) demonstrate correct detection using the proposed methods.

### Database of AD

**Fig 5:**
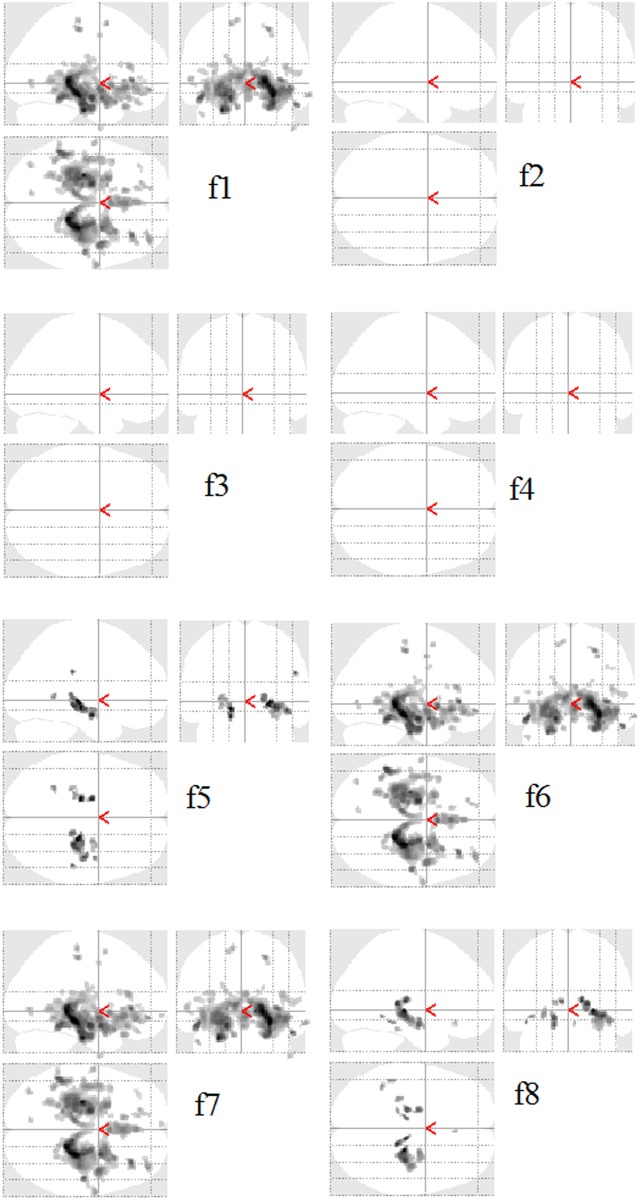
Statistical map of the local textures corrected by FDR at p<0.01 in the AD database. VGLCM-TOP-3D at Q = 8 was used to compute the features.

Since VGLCM-TOP-3D provided a better performance with detection of artificial lesions, VGLCM-TOP-3D at Q = 8 was used to study cerebral changes in AD. To decrease the false positive error FDR was set at p<0.01 instead of p<0.05, and only clusters with at least 10 voxel extensions were considered in the generation of statistical maps. Differences in AD in all textures except f2, f3, and f4 were found (Fig. 5, Table 3). The bulk of the findings are concentrated in the medial temporal lobes. The results of f5 and f8 and the results of f1, f6, and f7 were similar. Features f1, f6, and f7 showed larger regions compared to f5 and f8. Table 3 summarizes the detected regions using voxel-based texture analysis in this paper and from other studies in AD.

**Table 3:** Regions detected by texture features and comparing that with regions reported by other studies.

Region Name	Laterality	Texture Features	Other studies
Anterior Cingulate	R	1,6,7,8	[23–26]
Corpus Callosum	L	1,6,7	[27–28]
Corpus Callosum	R	1,6,7	[27–28]
Hippocampus	R	1,5,6,7,8	[23–24]
Hippocampus	L	1,5,6,7,8	[23–24]
Inferior Frontal Gyrus	R	1,6,7	[25], [29–30]
Inferior Frontal Gyrus	L	1,7	[25–26], [30]
Inferior Parietal Lobule	R	5	[25], [31]
Insula	L	1,6,7	[23–25], [31]
Insula	R	1,6,7	[23–24]
Medial Frontal Gyrus	R	1,6,7,8	[24], [32]
Medial Frontal Gyrus	L	1,6,7	[24–26], [29]
Middle Frontal Gyrus	R	1,6,7	[25–26], [29]
Middle Frontal Gyrus	L	1,6,7	[25–26], [29–30]
Midbrain	L	1,5,6,7,8	[31], [33–34]
Parahippocampal Gyrus	R	1,5,6,7,8	[23], [29]
Parahippocampal Gyrus	L	1,5,6,7,8	[23], [29]
Precentral Gyrus	R	1,6,7	[30], [35–36]
Superior Frontal Gyrus	L	7	[30]
Temporal Lobe	L	1,5,6,7,8	[23], [26], [29–31]
Temporal Lobe	R	1,5,6,7,8	[23], [26], [29–31]
Thalamus	L	1,5,6,7,8	[23], [29], [31]
Thalamus	R	1,5,6,7,8	[23], [29], [31]

## Discussion

In this paper a novel method for 3D voxel-based texture analysis was presented. While the current approach of ROI-based texture analysis has been successfully used in several applications such as characterization of brain tumors [2–3], detection of lesions in epilepsy [4–6] and multiple sclerosis [9–11], and to study AD [7–8], it is limited to the analysis of a specified anatomical region. To the best of our knowledge, there is no spatially non-specific texture analysis method that provides a 3D statistical map. The most similar approach to our method was performed by Bernasconi et al. [4]. Their work was primarily based on first-order texture analysis (i.e., GM thickness, gradient, relative intensity), which computes the ratio map (i.e., ratio map = (GM thickness × relative intensity)/gray level intensity gradient). In the approach presented in this paper, however, a second order texture statistic (co-occurrence matrix) was used which is more precise and accurate than the first order statistic. Moreover, the output format of our method is a statistical map similar to that provided by VBM.

In general, the proposed VGLCM-TOP-3D has a higher performance compared to that of VGLCM-3D. It is because the approach that VGLCM-TOP-3D uses to extract 3D information is more sensitive to subtle changes occurring at edges. This is illustrated in S3 Fig. In this example, the value of autocorrelation (f1) using distance D = 1 and neighboring radius R = 1 was computed and it is explained how the two methods distinguish an edge that appears in the sagittal view (without loss of generality the edge can be considered in the other directions). Consider two neighboring voxels (blue and red) located on an edge. Assume that the blue and white voxels are located on the bright side of the edge with a gray value of 8 and the red and gray voxels are located on the dark side of the edge with a gray value of 1. As can be seen when three orthogonal planes are considered at the red/blue voxels, the edge appears in the axial and coronal planes while from the sagittal view the blue and red voxels are located in the pure bright and dark regions, respectively. As a result, the difference of the texture values (e.g. f1) is remarkably different in the sagittal plane. This difference appears in the final texture value which is the average of the texture values at the three planes. On the other hand, VGLCM-3D considers all directions. As one can see, considering all directions results in a higher difference of the texture feature (f1) compared to the coronal and axial views. However, the final feature difference of VGLCM-TOP-3D is higher because of the high difference of f1 in the sagittal view. In other words, since three different directions are considered in VGLCM-TOP-3D an edge makes a remarkable difference in at least one of the planes which results in a higher difference in the final feature values of the voxels located around the edge. This sensitivity to edges makes VGLCM-TOP-3D more discriminative than VGLCM-3D. It can be observed in Fig. 2 that the VGLCM-3D features are more blur than VGLCM-TOP-3D (it is more evident in f2, f3, f5, and f8).

The results on the artificial effects database show that the method correctly detects artificial effects even if they are small in size. The detection of lesions is based on the comparison of texture features at each voxel, whereby texture features are computed for each voxel and then the resulting texture maps undergone a voxel-wise statistical analysis. Since lesions have different texture features compared to corresponding non-lesion regions (the original images) they appear by statistical significance in the statistical map shown in Fig. 4.

In general, the texture features had a higher performance on hypo-intense (Types I, III, V, VII) compared to hyper-intense (Types II, IV, VI, VIII) artificial lesions for T1-weighted images (Tables 1 and 2).

The results on the AD database showed differences in textures between patients and controls that were most prominent in the medial temporal lobe. The spatial distribution of these changes is the same as the distribution of the pathological changes in AD. This, along with the concordance of our findings with other imaging studies in AD (Table 3), provides clinical relevance and validity to the proposed voxel-based texture analysis.

There are several choices of parameters when one performs local texture analysis, which include the number of gray levels, the neighborhood size, and the offset distance. Since texture features are computed in a small spherical region around voxels, a small value of gray levels (G) is enough to get good results (e.g., 8 or 16). Also, a change of G has negligible impact on the performance of the methods particularly for VGLCM-TOP-3D. For VGLCM-3D, increasing the number of gray levels reduces the FN error while it raises the FP error. The neighborhood radius (*R*) and the offset distance (*D*) should be large enough to be able to distinguish texture patterns, while small enough to detect local changes around each voxel. A value of 1 to 3 is a good choice for *R* and *D*.

The proposed methods do not require a pre-defined a region of interest for analysis as they provide a hypothesis-free analysis tool to detect regions affected by a disease; as such the method is more easily translatable to clinical practice.

### Advantages

Important properties of texture analysis that make it advantageous for use in MRI analysis includes robustness with respect to acquisition parameters, such as the number of averages, repetition time, echo time, and sampling bandwidth [12]. Moreover, recent texture methods [13–15] also demonstrate robustness to noise.

ROI-based texture analysis methods require segmentation. Accurate segmentation may best be achieved when performed manually and this could become the bottleneck of the processing pipeline, as was the case, for instance, in the work of De Oliveira et al. [8] where relatively simple and easily segmented structures (corpus callosum and thalamus) were studied in AD. The proposed method in this paper obviates the need for segmentation as it performs analysis on a voxel-by-voxel basis in the whole brain.

The presented tool can be incorporated into current popular brain imaging analysis software packages such as SPM and FSL and is a complementary method to VBM.

A significant advantage of the proposed method is that it does not need segmentation. In contrast, VBM requires accurate segmentation of WM and GM as the analysis is done only on one tissue type and thus VBM needs to address the confounding issue of partial volume averaging (when a voxel contains both WM and GM). The presented texture method does not need segmentation, and therefore, reduces the computational complexity of segmentation as well as errors associated with segmentation inaccuracies. Moreover, texture analysis is not restricted to GM or WM and thus would be attractive to the study of neuropsychiatric disorders that include pathology in both tissue classes. Furthermore, VBM detects reduced tissue density and is not sensitive to other structural changes (e.g., shape around each voxel), while texture analysis can detect more complex structural changes. Nonetheless, a comprehensive study between the two methods should be performed to compare the advantages of each method.

### Limitations and Future Works

While texture analysis provides useful information it requires additional computations to process data. The average running time to compute texture features for a subject on a typical PC with an Intel quad core 2.60 GHz CPU with 16GB RAM running Windows 7 Professional is about 15 minutes for VGLCM-3D with Q = 8, 33 minutes for VGLCM-3D with Q = 16, 14 minutes for VGLCM-TOP-3D with Q = 8, and 20 minutes for VGLCM-TOP-3D with Q = 16.

It should be noted that the pattern, strength, and spatial extent of hypo/hyper- intense lesions on brain images are different from disease to disease and therefore for each neurological disease a different set of texture features might be useful. For instance, in the AD database f2, f3, and f4 did not reveal statistical difference after FDR at p<0.01, and f5, and f8 showed a smaller region compared to f1, f6, and f7. As a result, our recommendation is to first do an exploratory analysis by computing all textures features. A combination of textures using methods such as discriminant analysis may be more robust.

The proposed method is based on the relatively established method of GLCM for texture computation. More novel texture analysis methods which are robust to noise and non-uniformity of intensity will be considered for future investigation. Finally, T1-weighted MRI was used to perform texture analysis as it is a standard MR image contrast acquired in neurological disorders. However, the proposed method can be extended without modification to probe texture-based signatures in MR images of other contrasts (e.g. T2-weighted).

## Conclusions

In this paper, a novel method for voxel-based 3D texture analysis was proposed as a powerful image analysis tool. The output is a statistical map comparable to that of VBM showing differences in textures rather than GM or WM density. The proposed analysis was tested successfully to evaluate artificial lesions and demonstrate cerebral changes in an MRI database of AD. The method could detect the artificial lesions accurately and the regions detected in the AD database were consistent with the known spatial pathological distribution of this disease. The proposed voxel based texture analysis shows promise as a tool to study neurological disorders *in vivo* and has the potential to be a biomarker to aid in diagnosis, monitor disease progression, and evaluate treatment. The proposed method has been implemented as a toolbox for SPM and can be used to study different diseases affecting the brain.

## Supporting Information

S1 Fig The location of artificial lesions. (TIF)

S2 Fig Illustration of a GLCM computation: (a) Vector with distance d and angle θ, (b) A sample image with pixels of a 4 x 4 sample image, three gray levels (L = 0,1,2), and GLCM vectors with d = 1 and θ = 90o, and (c) The resulting GLCM. (TIF)

S3 Fig Computing f1 by VGLCM-TOP-3D and VGLCM-3D methods for a voxel located on a sagittal edge (i.e., between dark and bright regions). Top left, the original image, bottom left VGLCM-3D, and right VGLCM-TOP-3D.
(TIF)

S1 Table Specifications of the artificial effects. (DOC)

S2 Table Texture features used in this paper and their formula: p is in the VGLCM-3D method (or, and in the VGLCM-TOP-3D method), and Ng the number of gray levels (quantization level). Auxiliary formulae are given in the bottom of the table.
(DOC)

S3 Table The performance of the best texture feature, f1 (Autocorrelation) computed for the 8 artificial effect types. Each artificial effect type consists of 60 artificial lesions. The statistical significance of quantization level is shown by ‡ and the statistical significance of method (VGLCM-TOP-3D vs VGLCM-3D) is shown by * (p<0.05).
(DOC)

S4 Table The performance of the best texture feature, f2 (Homogeneity) computed for the 8 artificial effect types. Each artificial effect type consists of 60 artificial lesions. The statistical significance of quantization level is shown by ‡ and the statistical significance of method (VGLCM-TOP-3D vs VGLCM-3D) is shown by * (p<0.05).
(DOC)

S5 Table The performance of the best texture feature, f3 (Energy) computed for the 8 artificial effect types. The statistical significance of quantization level is shown by ‡ and the statistical significance of method (VGLCM-TOP-3D vs VGLCM-3D) is shown by * (p<0.05).
(DOC)

S6 Table The performance of the best texture feature, f4 (Correlation) computed for the 8 artificial effect types. The statistical significance of quantization level is shown by ‡ and the statistical significance of method (VGLCM-TOP-3D vs VGLCM-3D) is shown by * (p<0.05).
(DOC)

S7 Table The performance of the best texture feature, f5 (Dissimilarity) computed for the 8 artificial effect types. The statistical significance of quantization level is shown by ‡ and the statistical significance of method (VGLCM-TOP-3D vs VGLCM-3D) is shown by * (p<0.05).
(DOC)

S8 Table The performance of the best texture feature, f7 (Sum average) computed for the 8 artificial effect types. The statistical significance of quantization level is shown by ‡ and the statistical significance of method (VGLCM-TOP-3D vs VGLCM-3D) is shown by * (p<0.05).
(DOC)

S9 Table The performance of the best texture feature, f8 (Sum entropy) computed for the 8 artificial effect types. The statistical significance of quantization level is shown by ‡ and the statistical significance of method (VGLCM-TOP-3D vs VGLCM-3D) is shown by * (p<0.05).
(DOC)
