# Supplementary figures and images for: Voxel-Based Texture Analysis of the Brain
Source: PLoS One. 2015 Mar 10;10(3):e0117759. doi: 10.1371/journal.pone.0117759 (PMC4355627; doi:10.1371/journal.pone.0117759)

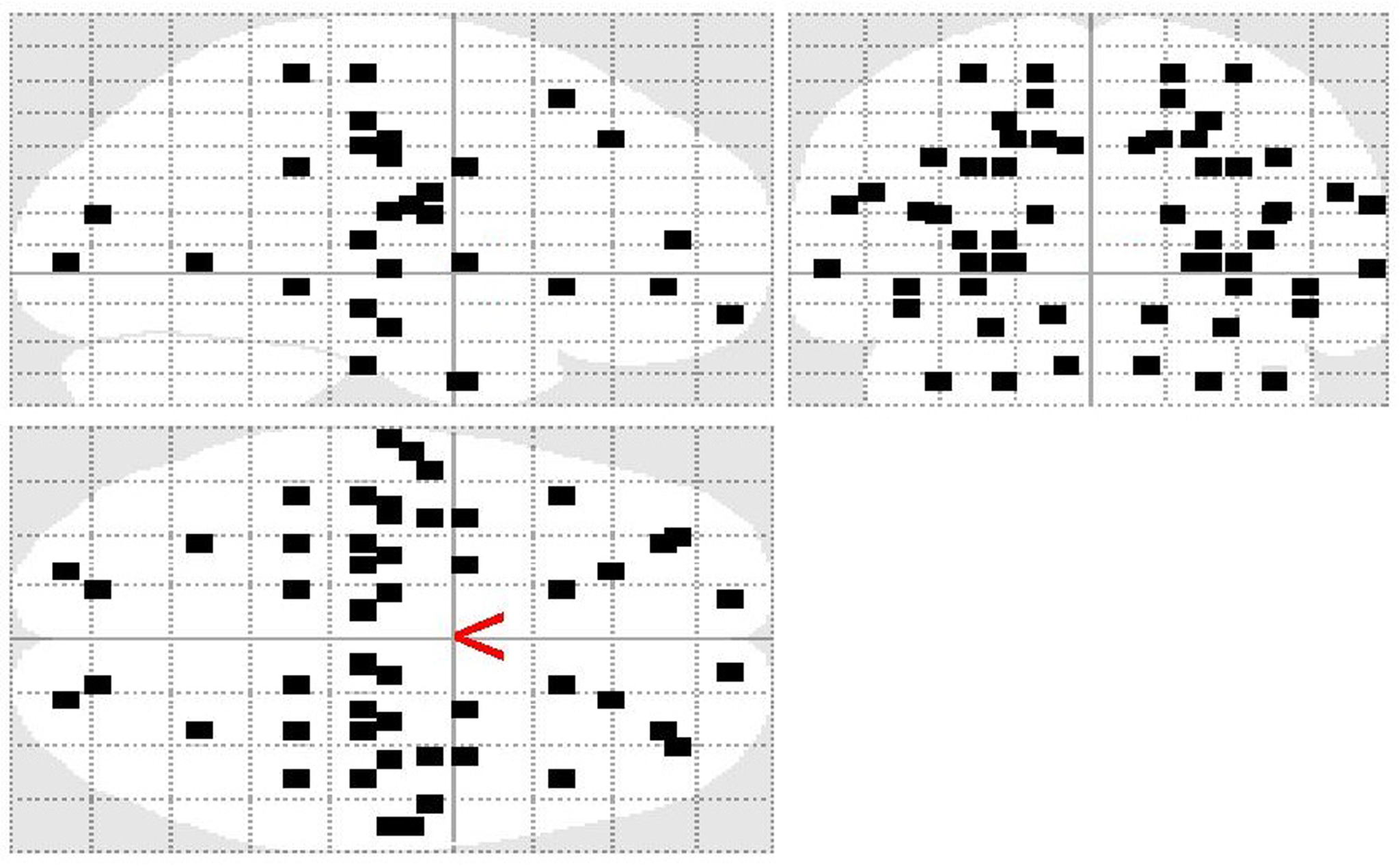

Supplement: S1 Fig — (TIF) [file pone.0117759.s001.tif]

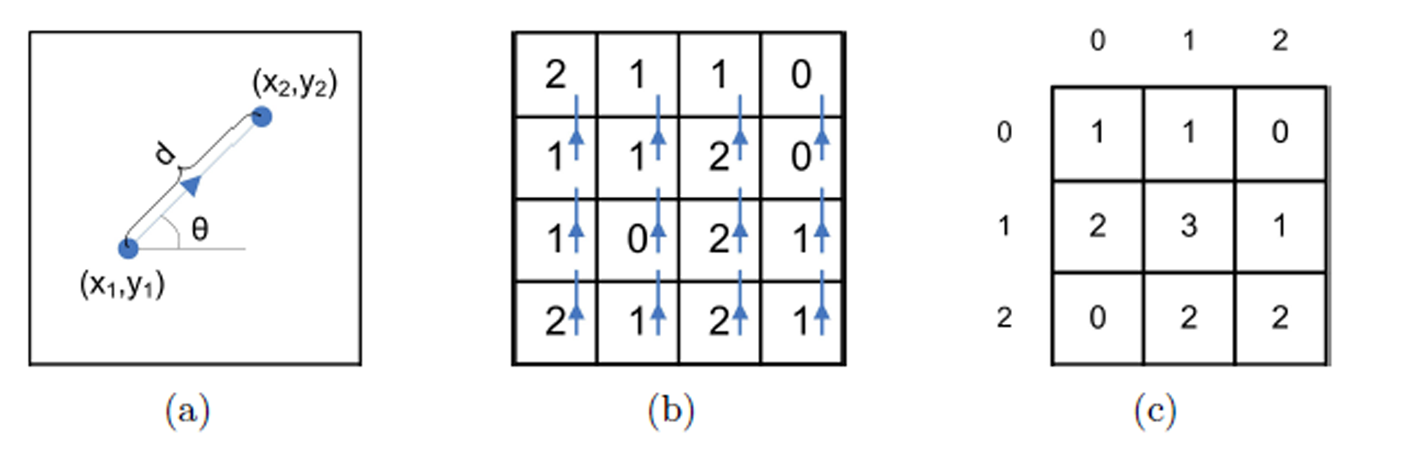

Supplement: S2 Fig — (TIF) [file pone.0117759.s002.tif]

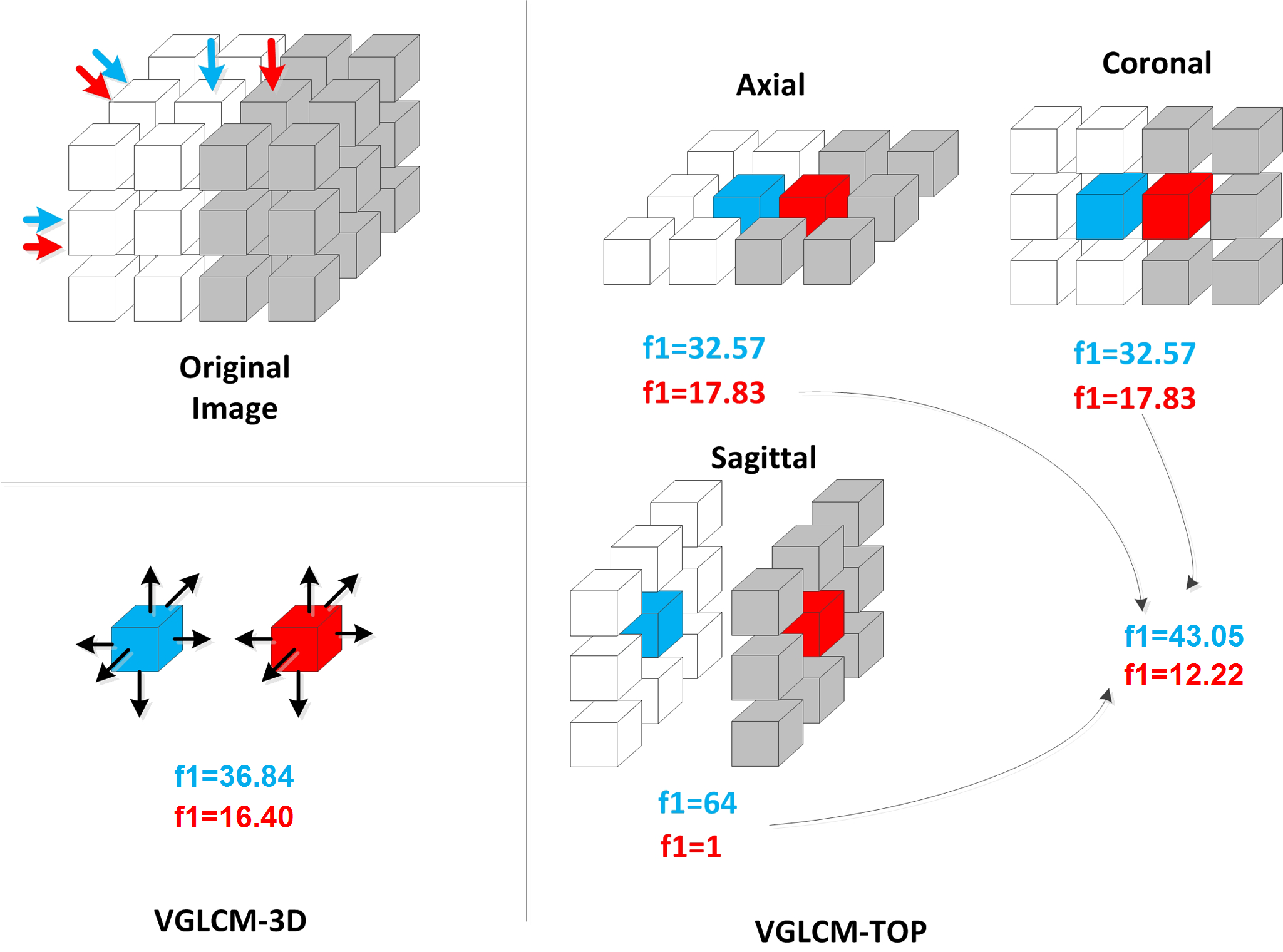

Supplement: S3 Fig — Top left, the original image, bottom left VGLCM-3D, and right VGLCM-TOP-3D. (TIF) [file pone.0117759.s003.tif]
