# Supplementary material for: Voxel-Based Texture Analysis of the Brain
Source: PLoS One. 2015 Mar 10;10(3):e0117759. doi: 10.1371/journal.pone.0117759 (PMC4355627; doi:10.1371/journal.pone.0117759)
Supplement: S1 Table — (DOC) [file pone.0117759.s004.doc]

Table S1. Specifications of the artificial effects.

| Artificial Effect | Hypo/Hyper –Intense | Size | Mean  of Gaussian | Standard deviation  of Gaussian |
| --- | --- | --- | --- | --- |
| Type I | Hypo-intense | 3 | 200 | 50 |
| Type II | Hyper-intense | 3 | 200 | 50 |
| Type III | Hypo-intense | 3 | 400 | 50 |
| Type IV | Hyper-intense | 3 | 400 | 50 |
| Type V | Hypo-intense | 4 | 200 | 50 |
| Type VI | Hyper-intense | 4 | 200 | 50 |
| Type VII | Hypo-intense | 4 | 400 | 50 |
| Type VIII | Hyper-intense | 4 | 400 | 50 |
