# Supplementary material for: Voxel-Based Texture Analysis of the Brain
Source: PLoS One. 2015 Mar 10;10(3):e0117759. doi: 10.1371/journal.pone.0117759 (PMC4355627; doi:10.1371/journal.pone.0117759)
Supplement: S2 Table — Auxiliary formulae are given in the bottom of the table. (DOC) [file pone.0117759.s005.doc]

Table S2. Texture features used in this paper and their formula: p is in the VGLCM-3D method (or , and in the VGLCM-TOP-3D method), and N_g_ the number of gray levels (quantization level). Auxiliary formulae are given in the bottom of the table.

|  | Texture Name | | Formula |
| --- | --- | --- | --- |
| f1 | Autocorrelation | |  |
| f2 | Homogeneity | |  |
| f3 | Energy | |  |
| f4 | Correlation | |  |
| f5 | Dissimilarity | |  |
| f6 | Sum of squares: variance | |  |
| f7 | Sum average | |  |
| f8 | Sum entropy | |  |
|  | |  | |
|  | |  | |
|  | |  | |
