# Supplementary material for: Voxel-Based Texture Analysis of the Brain
Source: PLoS One. 2015 Mar 10;10(3):e0117759. doi: 10.1371/journal.pone.0117759 (PMC4355627; doi:10.1371/journal.pone.0117759)
Supplement: S3 Table — Each artificial effect type consists of 60 artificial lesions. The statistical significance of quantization level is shown by ‡ and the statistical significance of method (VGLCM-TOP-3D vs VGLCM-3D) is shown by * (p<0.05). (DOC) [file pone.0117759.s006.doc]

Table S3. The performance of the best texture feature, f1 (Autocorrelation) computed for the 8 artificial effect types. Each artificial effect type consists of 60 artificial lesions. The statistical significance of quantization level is shown by ‡ and the statistical significance of method (VGLCM-TOP-3D vs VGLCM-3D) is shown by * (p<0.05).

|  |  | Q= 8 | | | | Q= 16 | | | |
| --- | --- | --- | --- | --- | --- | --- | --- | --- | --- |
|  | Type | Detect | UO | FN Error | FP Error | Detect | UO | FN Error | FP Error |
| VGLCM-TOP-3D | I | 98% | 0.67±0.26 | 0.20±0.29 | 0.14±0.17 | 98% | 0.63±0.23 | 0.17±0.26 | 0.21±0.20 |
|  | II | 92% | 0.53±0.28 | 0.42±0.31 | 0.06±0.11 | 95% | 0.64±0.27 | 0.24±0.31 | 0.13±0.18 |
|  | III | 100% | 0.51±0.17 | 0.01±0.03 | 0.48±0.19 | 100% | 0.48±0.18 | 0.01±0.04 | 0.51±0.20 |
|  | IV | 100% | 0.58±0.16 | 0.01±0.03 | 0.42±0.18 | 100% | 0.62±0.18 | 0.02±0.03 | 0.37±0.19 |
|  | V | 100% | 0.71±0.16 | 0.12±0.19 | 0.18±0.15 | 100% | 0.66±0.15 | 0.10±0.18 | 0.26±0.16 |
|  | VI | 100% | 0.74±0.19 | 0.17±0.21 | 0.10±0.12 | 100% | 0.72±0.17 | 0.11±0.19 | 0.18±0.15 |
|  | VII | 100% | 0.53±0.16 | 0.00±0.01 | 0.47±0.17 | 100% | 0.50±0.17 | 0.00±0.01 | 0.50±0.18 |
|  | VIII | 100% | 0.65±0.15 | 0.00±0.01 | 0.35±0.16 | 100% | 0.68±0.16 | 0.01±0.01 | 0.32±0.17 |
|  | ALL | 99% | 0.61±0.21* | 0.08±0.19* | 0.31±0.22*‡ | 99% | 0.61±0.21* | 0.08±0.19*‡ | 0.31±0.22* |
| VGLCM-3D | I | 98% | 0.60±0.25 | 0.23±0.29 | 0.18±0.20 | 98% | 0.55±0.24 | 0.21±0.29 | 0.26±0.22 |
|  | II | 90% | 0.42±0.27 | 0.53±0.31 | 0.06±0.12 | 93% | 0.56±0.26 | 0.30±0.32 | 0.15±0.19 |
|  | III | 100% | 0.43±0.16 | 0.02±0.05 | 0.56±0.19 | 100% | 0.40±0.16 | 0.02±0.06 | 0.58±0.19 |
|  | IV | 100% | 0.51±0.17 | 0.03±0.08 | 0.47±0.20 | 100% | 0.57±0.20 | 0.04±0.08 | 0.40±0.23 |
|  | V | 100% | 0.61±0.15 | 0.15±0.22 | 0.27±0.17 | 100% | 0.55±0.13 | 0.13±0.21 | 0.35±0.18 |
|  | VI | 100% | 0.66±0.18 | 0.22±0.23 | 0.14±0.14 | 100% | 0.63±0.17 | 0.15±0.21 | 0.24±0.17 |
|  | VII | 100% | 0.45±0.12 | 0.01±0.02 | 0.55±0.13 | 100% | 0.43±0.13 | 0.01±0.03 | 0.56±0.14 |
|  | VIII | 100% | 0.56±0.14 | 0.01±0.03 | 0.44±0.16 | 100% | 0.59±0.15 | 0.02±0.03 | 0.40±0.17 |
|  | ALL | 98% | 0.52±0.21 | 0.16±0.26 | 0.33±0.25‡ | 99% | 0.53±0.20 | 0.11±0.22‡ | 0.37±0.23 |
