# Supplementary material for: Voxel-Based Texture Analysis of the Brain
Source: PLoS One. 2015 Mar 10;10(3):e0117759. doi: 10.1371/journal.pone.0117759 (PMC4355627; doi:10.1371/journal.pone.0117759)
Supplement: S4 Table — Each artificial effect type consists of 60 artificial lesions. The statistical significance of quantization level is shown by ‡ and the statistical significance of method (VGLCM-TOP-3D vs VGLCM-3D) is shown by * (p<0.05). (DOC) [file pone.0117759.s007.doc]

Table S4. The performance of the best texture feature, f2 (Homogeneity) computed for the 8 artificial effect types. Each artificial effect type consists of 60 artificial lesions. The statistical significance of quantization level is shown by ‡ and the statistical significance of method (VGLCM-TOP-3D vs VGLCM-3D) is shown by * (p<0.05).

|  |  | Q= 8 | | | | Q= 16 | | | |
| --- | --- | --- | --- | --- | --- | --- | --- | --- | --- |
|  | Type | Detect | UO | FN Error | FP Error | Detect | UO | FN Error | FP Error |
| VGLCM-TOP-3D | I | 100% | 0.44±0.19 | 0.29±0.29 | 0.39±0.22 | 100% | 0.44±0.15 | 0.21±0.25 | 0.43±0.22 |
|  | II | 88% | 0.45±0.27 | 0.42±0.36 | 0.20±0.23 | 90% | 0.41±0.23 | 0.31±0.38 | 0.33±0.25 |
|  | III | 100% | 0.34±0.09 | 0.03±0.11 | 0.66±0.09 | 100% | 0.36±0.12 | 0.05±0.13 | 0.63±0.14 |
|  | IV | 100% | 0.34±0.10 | 0.06±0.18 | 0.64±0.10 | 100% | 0.41±0.12 | 0.15±0.24 | 0.49±0.19 |
|  | V | 100% | 0.39±0.12 | 0.40±0.23 | 0.39±0.20 | 100% | 0.41±0.10 | 0.28±0.24 | 0.43±0.20 |
|  | VI | 100% | 0.44±0.17 | 0.30±0.31 | 0.36±0.17 | 100% | 0.40±0.15 | 0.31±0.30 | 0.41±0.19 |
|  | VII | 100% | 0.37±0.07 | 0.07±0.12 | 0.61±0.07 | 100% | 0.39±0.09 | 0.09±0.15 | 0.59±0.10 |
|  | VIII | 100% | 0.36±0.07 | 0.09±0.17 | 0.62±0.06 | 100% | 0.36±0.10 | 0.30±0.25 | 0.54±0.13 |
|  | ALL | 98% | 0.39±0.16* | 0.21±0.28 | 0.48±0.23* | 99% | 0.39±0.14* | 0.21±0.27 | 0.48±0.20* |
| VGLCM-3D | I | 97% | 0.39±0.13 | 0.28±0.28 | 0.45±0.20 | 97% | 0.39±0.14 | 0.22±0.27 | 0.49±0.24 |
|  | II | 87% | 0.37±0.23 | 0.45±0.37 | 0.27±0.26 | 90% | 0.31±0.16 | 0.33±0.40 | 0.41±0.28 |
|  | III | 100% | 0.30±0.06 | 0.02±0.05 | 0.70±0.06 | 100% | 0.31±0.08 | 0.02±0.06 | 0.68±0.09 |
|  | IV | 98% | 0.29±0.08 | 0.07±0.20 | 0.67±0.13 | 98% | 0.34±0.11 | 0.11±0.25 | 0.59±0.17 |
|  | V | 100% | 0.35±0.10 | 0.37±0.24 | 0.50±0.15 | 100% | 0.38±0.08 | 0.26±0.23 | 0.52±0.13 |
|  | VI | 96% | 0.39±0.12 | 0.26±0.31 | 0.46±0.17 | 98% | 0.37±0.11 | 0.25±0.30 | 0.50±0.15 |
|  | VII | 100% | 0.32±0.05 | 0.04±0.11 | 0.67±0.05 | 100% | 0.33±0.05 | 0.05±0.10 | 0.66±0.05 |
|  | VIII | 100% | 0.32±0.06 | 0.06±0.15 | 0.67±0.07 | 100% | 0.31±0.09 | 0.22±0.26 | 0.63±0.09 |
|  | ALL | 97% | 0.34±0.13 | 0.20±0.29 | 0.54±0.21 | 98% | 0.34±0.11 | 0.18±0.28 | 0.56±0.19 |
