# Supplementary material for: Voxel-Based Texture Analysis of the Brain
Source: PLoS One. 2015 Mar 10;10(3):e0117759. doi: 10.1371/journal.pone.0117759 (PMC4355627; doi:10.1371/journal.pone.0117759)
Supplement: S5 Table — The statistical significance of quantization level is shown by ‡ and the statistical significance of method (VGLCM-TOP-3D vs VGLCM-3D) is shown by * (p<0.05). (DOC) [file pone.0117759.s008.doc]

Table S5. The performance of the best texture feature, f3 (Energy) computed for the 8 artificial effect types. The statistical significance of quantization level is shown by ‡ and the statistical significance of method (VGLCM-TOP-3D vs VGLCM-3D) is shown by * (p<0.05).

|  |  | Q= 8 | | | | Q= 16 | | | |
| --- | --- | --- | --- | --- | --- | --- | --- | --- | --- |
|  | Type | Detect | UO | FN Error | FP Error | Detect | UO | FN Error | FP Error |
| VGLCM-TOP-3D | I | 100% | 0.43±0.15 | 0.32±0.27 | 0.38±0.20 | 100% | 0.44±0.15 | 0.30±0.31 | 0.34±0.20 |
|  | II | 83% | 0.36±0.25 | 0.52±0.35 | 0.23±0.24 | 87% | 0.36±0.23 | 0.41±0.41 | 0.29±0.21 |
|  | III | 100% | 0.43±0.11 | 0.07±0.12 | 0.55±0.12 | 100% | 0.48±0.15 | 0.17±0.26 | 0.42±0.16 |
|  | IV | 100% | 0.42±0.10 | 0.08±0.16 | 0.55±0.13 | 93% | 0.35±0.17 | 0.43±0.34 | 0.39±0.18 |
|  | V | 100% | 0.37±0.11 | 0.44±0.22 | 0.40±0.18 | 100% | 0.42±0.15 | 0.35±0.31 | 0.35±0.16 |
|  | VI | 100% | 0.41±0.17 | 0.35±0.32 | 0.39±0.14 | 98% | 0.36±0.18 | 0.45±0.34 | 0.38±0.17 |
|  | VII | 100% | 0.44±0.08 | 0.12±0.12 | 0.52±0.09 | 100% | 0.48±0.13 | 0.20±0.25 | 0.42±0.14 |
|  | VIII | 100% | 0.41±0.09 | 0.15±0.16 | 0.55±0.10 | 98% | 0.35±0.15 | 0.48±0.27 | 0.40±0.18 |
|  | ALL | 98% | 0.41±0.15* | 0.26±0.28‡ | 0.44±0.19* | 97% | 0.41±0.18* | 0.35±0.33 | 0.37±0.18*‡ |
| VGLCM-3D | I | 100% | 0.34±0.12 | 0.40±0.29 | 0.43±0.22 | 97% | 0.35±0.13 | 0.35±0.34 | 0.41±0.25 |
|  | II | 88% | 0.29±0.20 | 0.57±0.33 | 0.35±0.25 | 92% | 0.28±0.16 | 0.40±0.41 | 0.39±0.27 |
|  | III | 100% | 0.34±0.07 | 0.12±0.14 | 0.63±0.09 | 100% | 0.37±0.09 | 0.17±0.24 | 0.57±0.13 |
|  | IV | 100% | 0.32±0.08 | 0.13±0.20 | 0.64±0.11 | 96% | 0.27±0.11 | 0.38±0.33 | 0.61±0.17 |
|  | V | 100% | 0.32±0.08 | 0.45±0.21 | 0.50±0.15 | 100% | 0.36±0.09 | 0.34±0.28 | 0.46±0.15 |
|  | VI | 100% | 0.34±0.12 | 0.35±0.33 | 0.49±0.16 | 96% | 0.30±0.11 | 0.40±0.32 | 0.53±0.17 |
|  | VII | 100% | 0.36±0.05 | 0.14±0.13 | 0.61±0.05 | 100% | 0.38±0.07 | 0.18±0.22 | 0.56±0.08 |
|  | VIII | 100% | 0.33±0.07 | 0.15±0.18 | 0.63±0.08 | 100% | 0.28±0.10 | 0.41±0.26 | 0.61±0.13 |
|  | ALL | 98% | 0.33±0.11 | 0.29±0.29 | 0.53±0.19 | 98% | 0.32±0.12 | 0.33±0.32 | 0.52±0.20 |
