# Supplementary material for: Voxel-Based Texture Analysis of the Brain
Source: PLoS One. 2015 Mar 10;10(3):e0117759. doi: 10.1371/journal.pone.0117759 (PMC4355627; doi:10.1371/journal.pone.0117759)
Supplement: S6 Table — The statistical significance of quantization level is shown by ‡ and the statistical significance of method (VGLCM-TOP-3D vs VGLCM-3D) is shown by * (p<0.05). (DOC) [file pone.0117759.s009.doc]

Table S6. The performance of the best texture feature, f4 (Correlation) computed for the 8 artificial effect types. The statistical significance of quantization level is shown by ‡ and the statistical significance of method (VGLCM-TOP-3D vs VGLCM-3D) is shown by * (p<0.05).

|  |  | Q= 8 | | | | Q= 16 | | | |
| --- | --- | --- | --- | --- | --- | --- | --- | --- | --- |
|  | Type | Detect | UO | FN Error | FP Error | Detect | UO | FN Error | FP Error |
| VGLCM-TOP-3D | I | 95% | 0.22±0.11 | 0.70±0.17 | 0.37±0.28 | 98% | 0.29±0.15 | 0.59±0.20 | 0.39±0.26 |
|  | II | 73% | 0.12±0.11 | 0.85±0.14 | 0.25±0.26 | 93% | 0.20±0.13 | 0.73±0.20 | 0.39±0.28 |
|  | III | 100% | 0.27±0.12 | 0.51±0.16 | 0.56±0.26 | 100% | 0.30±0.14 | 0.42±0.20 | 0.59±0.23 |
|  | IV | 100% | 0.27±0.09 | 0.51±0.18 | 0.55±0.22 | 98% | 0.30±0.15 | 0.49±0.23 | 0.53±0.24 |
|  | V | 96% | 0.20±0.11 | 0.71±0.18 | 0.47±0.22 | 100% | 0.23±0.13 | 0.65±0.20 | 0.48±0.22 |
|  | VI | 95% | 0.19±0.11 | 0.76±0.15 | 0.35±0.21 | 98% | 0.26±0.11 | 0.64±0.19 | 0.40±0.21 |
|  | VII | 100% | 0.24±0.07 | 0.52±0.19 | 0.59±0.20 | 100% | 0.27±0.09 | 0.47±0.18 | 0.60±0.19 |
|  | VIII | 100% | 0.27±0.08 | 0.49±0.19 | 0.57±0.14 | 100% | 0.31±0.09 | 0.49±0.16 | 0.50±0.18 |
|  | ALL | 95% | 0.22±0.11* | 0.63±0.21 | 0.46±0.26* | 98% | 0.26±0.13*‡ | 0.56±0.22‡ | 0.48±0.24* |
| VGLCM-3D | I | 98% | 0.21±0.09 | 0.65±0.20 | 0.49±0.25 | 100% | 0.27±0.13 | 0.48±0.25 | 0.59±0.21 |
|  | II | 82% | 0.12±0.09 | 0.84±0.12 | 0.37±0.32 | 97% | 0.22±0.12 | 0.63±0.21 | 0.54±0.22 |
|  | III | 100% | 0.23±0.11 | 0.41±0.17 | 0.72±0.14 | 100% | 0.20±0.08 | 0.30±0.22 | 0.78±0.09 |
|  | IV | 100% | 0.23±0.09 | 0.49±0.21 | 0.65±0.18 | 100% | 0.25±0.10 | 0.39±0.23 | 0.66±0.18 |
|  | V | 98% | 0.20±0.10 | 0.65±0.21 | 0.59±0.17 | 100% | 0.23±0.08 | 0.54±0.22 | 0.63±0.13 |
|  | VI | 100% | 0.18±0.09 | 0.74±0.15 | 0.49±0.22 | 98% | 0.26±0.09 | 0.55±0.18 | 0.54±0.16 |
|  | VII | 100% | 0.23±0.05 | 0.40±0.17 | 0.70±0.10 | 100% | 0.23±0.06 | 0.27±0.14 | 0.75±0.08 |
|  | VIII | 100% | 0.26±0.08 | 0.45±0.19 | 0.64±0.11 | 100% | 0.29±0.09 | 0.35±0.16 | 0.63±0.15 |
|  | ALL | 97% | 0.20±0.10 | 0.59±0.24* | 0.58±0.23‡ | 99% | 0.24±0.10‡ | 0.44±0.24*‡ | 0.64±0.18 |
