# Supplementary material for: Voxel-Based Texture Analysis of the Brain
Source: PLoS One. 2015 Mar 10;10(3):e0117759. doi: 10.1371/journal.pone.0117759 (PMC4355627; doi:10.1371/journal.pone.0117759)
Supplement: S7 Table — The statistical significance of quantization level is shown by ‡ and the statistical significance of method (VGLCM-TOP-3D vs VGLCM-3D) is shown by * (p<0.05). (DOC) [file pone.0117759.s010.doc]

Table S7. The performance of the best texture feature, f5 (Dissimilarity) computed for the 8 artificial effect types. The statistical significance of quantization level is shown by ‡ and the statistical significance of method (VGLCM-TOP-3D vs VGLCM-3D) is shown by * (p<0.05).

|  |  | Q= 8 | | | | Q= 16 | | | |
| --- | --- | --- | --- | --- | --- | --- | --- | --- | --- |
|  | Type | Detect | UO | FN Error | FP Error | Detect | UO | FN Error | FP Error |
| VGLCM-TOP-3D | I | 97% | 0.37±0.20 | 0.40±0.30 | 0.48±0.24 | 98% | 0.36±0.17 | 0.28±0.30 | 0.53±0.22 |
|  | II | 85% | 0.38±0.27 | 0.52±0.35 | 0.24±0.27 | 92% | 0.38±0.23 | 0.35±0.38 | 0.40±0.26 |
|  | III | 98% | 0.26±0.10 | 0.17±0.28 | 0.73±0.09 | 100% | 0.24±0.08 | 0.10±0.23 | 0.75±0.07 |
|  | IV | 96% | 0.26±0.11 | 0.20±0.32 | 0.72±0.09 | 100% | 0.31±0.12 | 0.15±0.29 | 0.66±0.12 |
|  | V | 100% | 0.33±0.13 | 0.53±0.20 | 0.43±0.20 | 100% | 0.36±0.13 | 0.35±0.25 | 0.50±0.20 |
|  | VI | 100% | 0.39±0.18 | 0.40±0.31 | 0.41±0.18 | 100% | 0.38±0.17 | 0.31±0.31 | 0.49±0.16 |
|  | VII | 100% | 0.31±0.09 | 0.18±0.25 | 0.67±0.07 | 100% | 0.29±0.08 | 0.11±0.21 | 0.70±0.07 |
|  | VIII | 100% | 0.30±0.09 | 0.20±0.27 | 0.68±0.06 | 100% | 0.32±0.10 | 0.21±0.25 | 0.64±0.09 |
|  | ALL | 97% | 0.33±0.17* | 0.33±0.32 | 0.54±0.24*‡ | 99% | 0.33±0.15* | 0.24±0.30‡ | 0.58±0.20* |
| VGLCM-3D | I | 97% | 0.34±0.14 | 0.36±0.29 | 0.50±0.20 | 95% | 0.32±0.13 | 0.27±0.30 | 0.57±0.20 |
|  | II | 85% | 0.34±0.23 | 0.48±0.38 | 0.28±0.27 | 90% | 0.32±0.18 | 0.35±0.38 | 0.47±0.26 |
|  | III | 100% | 0.23±0.06 | 0.09±0.20 | 0.77±0.06 | 100% | 0.21±0.06 | 0.06±0.17 | 0.79±0.06 |
|  | IV | 98% | 0.25±0.09 | 0.13±0.27 | 0.71±0.13 | 100% | 0.28±0.10 | 0.11±0.25 | 0.69±0.13 |
|  | V | 100% | 0.30±0.10 | 0.47±0.23 | 0.55±0.14 | 100% | 0.31±0.10 | 0.34±0.26 | 0.59±0.12 |
|  | VI | 96% | 0.37±0.13 | 0.32±0.30 | 0.48±0.16 | 98% | 0.36±0.13 | 0.25±0.31 | 0.54±0.14 |
|  | VII | 100% | 0.26±0.06 | 0.12±0.22 | 0.73±0.05 | 100% | 0.25±0.06 | 0.07±0.18 | 0.75±0.06 |
|  | VIII | 100% | 0.27±0.07 | 0.13±0.24 | 0.71±0.06 | 100% | 0.30±0.09 | 0.12±0.25 | 0.67±0.07 |
|  | ALL | 97% | 0.30±0.13 | 0.27±0.31* | 0.59±0.22‡ | 98% | 0.30±0.12 | 0.20±0.29‡ | 0.63±0.18 |
