# Supplementary material for: Voxel-Based Texture Analysis of the Brain
Source: PLoS One. 2015 Mar 10;10(3):e0117759. doi: 10.1371/journal.pone.0117759 (PMC4355627; doi:10.1371/journal.pone.0117759)
Supplement: S8 Table — The statistical significance of quantization level is shown by ‡ and the statistical significance of method (VGLCM-TOP-3D vs VGLCM-3D) is shown by * (p<0.05). (DOC) [file pone.0117759.s011.doc]

Table S8. The performance of the best texture feature, f7 (Sum average) computed for the 8 artificial effect types. The statistical significance of quantization level is shown by ‡ and the statistical significance of method (VGLCM-TOP-3D vs VGLCM-3D) is shown by * (p<0.05).

|  |  | Q= 8 | | | | Q= 16 | | | |
| --- | --- | --- | --- | --- | --- | --- | --- | --- | --- |
|  | Type | Detect | UO | FN Error | FP Error | Detect | UO | FN Error | FP Error |
| VGLCM-TOP-3D | I | 98% | 0.67±0.26 | 0.22±0.29 | 0.13±0.16 | 98% | 0.61±0.23 | 0.19±0.28 | 0.22±0.19 |
|  | II | 90% | 0.52±0.28 | 0.45±0.31 | 0.05±0.10 | 95% | 0.62±0.27 | 0.27±0.31 | 0.12±0.17 |
|  | III | 100% | 0.51±0.17 | 0.01±0.03 | 0.49±0.18 | 100% | 0.48±0.18 | 0.01±0.02 | 0.51±0.19 |
|  | IV | 100% | 0.60±0.17 | 0.01±0.03 | 0.39±0.19 | 100% | 0.64±0.19 | 0.03±0.05 | 0.34±0.21 |
|  | V | 100% | 0.70±0.17 | 0.12±0.20 | 0.19±0.15 | 100% | 0.65±0.15 | 0.11±0.19 | 0.26±0.16 |
|  | VI | 100% | 0.73±0.19 | 0.19±0.22 | 0.09±0.11 | 100% | 0.72±0.17 | 0.13±0.20 | 0.16±0.14 |
|  | VII | 100% | 0.53±0.16 | 0.00±0.01 | 0.47±0.16 | 100% | 0.50±0.17 | 0.00±0.01 | 0.50±0.17 |
|  | VIII | 100% | 0.67±0.16 | 0.01±0.01 | 0.32±0.16 | 100% | 0.71±0.17 | 0.01±0.02 | 0.28±0.18 |
|  | ALL | 98% | 0.62±0.22* | 0.13±0.24* | 0.26±0.22*‡ | 99% | 0.62±0.21* | 0.10±0.20*‡ | 0.30±0.22* |
| VGLCM-3D | I | 95% | 0.58±0.25 | 0.26±0.30 | 0.18±0.20 | 97% | 0.54±0.23 | 0.23±0.30 | 0.26±0.22 |
|  | II | 88% | 0.41±0.26 | 0.55±0.31 | 0.06±0.12 | 93% | 0.53±0.25 | 0.34±0.32 | 0.15±0.19 |
|  | III | 100% | 0.42±0.16 | 0.02±0.06 | 0.56±0.18 | 100% | 0.41±0.16 | 0.02±0.06 | 0.58±0.18 |
|  | IV | 100% | 0.53±0.17 | 0.04±0.09 | 0.44±0.21 | 100% | 0.58±0.19 | 0.06±0.10 | 0.38±0.23 |
|  | V | 100% | 0.60±0.15 | 0.16±0.22 | 0.27±0.17 | 100% | 0.54±0.14 | 0.15±0.22 | 0.35±0.18 |
|  | VI | 100% | 0.64±0.19 | 0.24±0.24 | 0.14±0.14 | 100% | 0.63±0.17 | 0.17±0.22 | 0.22±0.17 |
|  | VII | 100% | 0.45±0.12 | 0.01±0.03 | 0.54±0.13 | 100% | 0.44±0.13 | 0.01±0.03 | 0.56±0.13 |
|  | VIII | 100% | 0.58±0.15 | 0.02±0.04 | 0.41±0.16 | 100% | 0.61±0.16 | 0.03±0.04 | 0.37±0.18 |
|  | ALL | 98% | 0.53±0.20 | 0.17±0.26 | 0.32±0.24‡ | 99% | 0.53±0.20 | 0.13±0.23‡ | 0.36±0.24 |
