# Supplementary material for: Voxel-Based Texture Analysis of the Brain
Source: PLoS One. 2015 Mar 10;10(3):e0117759. doi: 10.1371/journal.pone.0117759 (PMC4355627; doi:10.1371/journal.pone.0117759)
Supplement: S9 Table — The statistical significance of quantization level is shown by ‡ and the statistical significance of method (VGLCM-TOP-3D vs VGLCM-3D) is shown by * (p<0.05). (DOC) [file pone.0117759.s012.doc]

Table S9. The performance of the best texture feature, f8 (Sum entropy) computed for the 8 artificial effect types. The statistical significance of quantization level is shown by ‡ and the statistical significance of method (VGLCM-TOP-3D vs VGLCM-3D) is shown by * (p<0.05).

|  |  | Q= 8 | | | | Q= 16 | | | |
| --- | --- | --- | --- | --- | --- | --- | --- | --- | --- |
|  | Type | Detect | UO | FN Error | FP Error | Detect | UO | FN Error | FP Error |
| VGLCM-TOP-3D | I | 100% | 0.34±0.14 | 0.46±0.23 | 0.48±0.20 | 100% | 0.36±0.10 | 0.36±0.25 | 0.48±0.19 |
|  | II | 93% | 0.31±0.18 | 0.54±0.31 | 0.37±0.22 | 100% | 0.32±0.15 | 0.42±0.35 | 0.42±0.23 |
|  | III | 100% | 0.32±0.09 | 0.20±0.24 | 0.66±0.08 | 100% | 0.34±0.09 | 0.15±0.20 | 0.63±0.09 |
|  | IV | 100% | 0.28±0.09 | 0.21±0.26 | 0.69±0.08 | 100% | 0.31±0.08 | 0.30±0.24 | 0.61±0.13 |
|  | V | 100% | 0.32±0.10 | 0.53±0.17 | 0.45±0.16 | 100% | 0.37±0.11 | 0.37±0.23 | 0.46±0.17 |
|  | VI | 100% | 0.34±0.13 | 0.41±0.29 | 0.50±0.13 | 100% | 0.33±0.10 | 0.40±0.27 | 0.51±0.13 |
|  | VII | 100% | 0.35±0.09 | 0.22±0.20 | 0.61±0.07 | 100% | 0.37±0.08 | 0.17±0.20 | 0.60±0.07 |
|  | VIII | 100% | 0.31±0.08 | 0.23±0.23 | 0.65±0.06 | 100% | 0.30±0.07 | 0.38±0.21 | 0.60±0.11 |
|  | ALL | 99% | 0.32±0.12* | 0.35±0.28 | 0.55±0.18* | 100% | 0.34±0.10*‡ | 0.32±0.27 | 0.54±0.17* |
| VGLCM-3D | I | 100% | 0.30±0.10 | 0.46±0.25 | 0.53±0.15 | 100% | 0.30±0.08 | 0.36±0.27 | 0.56±0.15 |
|  | II | 93% | 0.26±0.14 | 0.48±0.35 | 0.52±0.21 | 100% | 0.24±0.08 | 0.39±0.35 | 0.59±0.20 |
|  | III | 100% | 0.27±0.04 | 0.17±0.16 | 0.71±0.05 | 100% | 0.27±0.05 | 0.14±0.15 | 0.72±0.06 |
|  | IV | 100% | 0.24±0.05 | 0.14±0.19 | 0.74±0.07 | 100% | 0.25±0.05 | 0.22±0.22 | 0.73±0.07 |
|  | V | 100% | 0.30±0.07 | 0.48±0.18 | 0.55±0.10 | 100% | 0.32±0.07 | 0.36±0.23 | 0.57±0.10 |
|  | VI | 100% | 0.29±0.09 | 0.34±0.30 | 0.61±0.09 | 100% | 0.28±0.07 | 0.34±0.27 | 0.63±0.09 |
|  | VII | 100% | 0.30±0.05 | 0.17±0.15 | 0.67±0.05 | 100% | 0.30±0.05 | 0.15±0.13 | 0.68±0.06 |
|  | VIII | 100% | 0.26±0.06 | 0.16±0.20 | 0.72±0.08 | 100% | 0.26±0.05 | 0.27±0.17 | 0.70±0.08 |
|  | ALL | 99% | 0.28±0.09 | 0.31±0.28* | 0.63±0.14 | 100% | 0.28±0.07 | 0.28±0.25* | 0.65±0.13 |
